# Electroreduction of divanillin to polyvanillin in an electrochemical flow reactor

**DOI:** 10.1186/s13065-024-01133-2

**Published:** 2024-02-08

**Authors:** Robin Kunkel, Maximilian Fath, Detlef Schmiedl, Volkmar M. Schmidt, Jens Tübke

**Affiliations:** 1 Department of Applied Electrochemistry, Fraunhofer Institute for Chemical Technology ICT, Joseph-Von-Fraunhofer-Str. 7, 76327 Pfinztal, Germany; 2 Institute of Chemical Process Engineering, Mannheim University of Applied Sciences, Paul-Wittsack-Str. 10, 68163 Mannheim, Germany; 3 Department of Environmental Engineering, Fraunhofer Institute for Chemical Technology ICT, Joseph-Von-Fraunhofer-Str. 7, 76327 Pfinztal, Germany

**Keywords:** Biobased polymers, Electroreduction, Electrosynthesis, Flow reactor, Polymerization, Vanillin

## Abstract

The electrochemical conversion of biobased intermediates offers an attractive and sustainable process for the production of green chemicals. One promising synthesis route is the production of the total vanillin-based polymer polyvanillin, which can be produced by electrochemical pinacolization of divanillin (5–5´bisvanillyl). Divanillin can be easily enzymatically generated from vanillin, a renewable intermediate accessible from lignin on an industrial scale. This study investigates systematically the electrochemical production of polyvanillin in a divided plane parallel flow reactor in recirculation mode. Several analytic methods, such as online UV–VIS spectroscopy, size exclusion chromatography (SEC), 2D-NMR (HSQC, ^13^C/^1^H), TGA and DSC were used to monitor the reaction progress and to characterize the reaction products under different galvanostatic reaction conditions revealing new insights into the reaction mechanism and structural features of the polymer. Further, by using an electrochemical engineering-based approach determining the limiting current densities, we readily achieved high current densities over 50 mA cm^−2^ for the polyvanillin synthesis and reached averaged molecular weights up to M_w_ = 4100 g mol^−1^ and M_n_ = 2700 g mol^−1^. The cathodic polymerization to polyvanillin offers an innovative approach for the electrochemical production of biobased polymers presented on flow cell level.

**Graphical Abstract:**

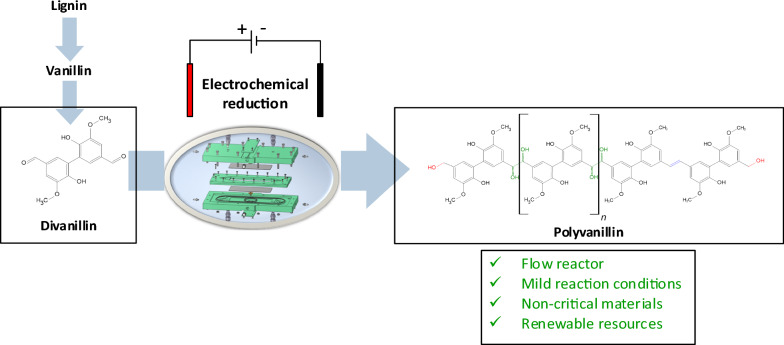

**Supplementary Information:**

The online version contains supplementary material available at 10.1186/s13065-024-01133-2.

## Introduction

The successive replacement of fossil-based polymers with polymers from renewable resources, so-called biobased polymers, is an effective way for the reduction of greenhouse gas emissions due to their carbon neutrality [1, 2]. Vanillin (4-hydroxy-3-methoxybenzaldehyde), a biobased platform chemical derived from lignin on an industrial scale, has therefore recently garnered increased attention as building block for biobased polymer synthesis [3]. Besides the traditional route to obtain vanillin from lignosulfonates via thermo-catalytic depolymerization in the presence of copper-based catalysts and oxygen operated by Borregaard (Norway) [4], many novel electrochemical strategies have been lately investigated achieving a total green process combining sustainable conversion technologies and renewable feedstock [5]. Different methods using either direct electrochemical oxidation [6, 7] or indirect oxidation via electrochemical generated oxidizer [8–10] were applied to obtain vanillin, vanillin acid and 5-Iodovanillin from lignin or lignosufonates. Many polymer synthesis strategies were reported making use of the multifunctional aromatic character of vanillin [11]. A wide range of vanillin-based polymers are accessible, such as phenolic [12], epoxy [13] and cyanate resins [14], polyesters [15] or polycarbonates [14].

A promising alternative to conventional synthesis routes to vanillin-based polymers and polymer building blocks is the reductive electrochemical pinacolization of vanillin’s carbonyl group enabling a sustainable pathway for C–C bond formation, since electrochemistry fulfills several criteria of the 12 principles of green chemistry [16]. Direct pinacolization of vanillin to hydrovanilloin was first described by I. A. Pearl in 1952 at Pb cathodes in diluted sodium hydroxide solution [17]. Due to its bisphenolic character hydrovanilloin was recently used by Amarasekara et al. for the synthesis of several polymers, such as a hydrovanilloin-formaldehyde polymer [18], a hydrovanilloin-diglycidyl ether phenoxy resin [19] or a poly(hydrovanilloin-urethane) [20]. Besides pinacolization transferring one electron to the carbonyl group followed by hydrodimerization, the carbonyl group can be reduced in a two-electron pathway to the corresponding alcohol. Jow et al. investigated the reaction mechanism of the vanillin reduction at Hg cathodes showing that pinacolization is favored over alcohol formation at higher pH-values, lower current densities and higher substrate concentrations. The reaction outcome is influenced by the deprotonation of vanillin’s phenolic group leading to a decreased stability of the negative charged intermediate species increasing the dimerization step over alcohol formation at higher pH values [21]. For reaching adequate faradaic efficiencies for the hydrovanilloin production cathode materials exhibiting a high overpotential for the competing hydrogen evolution reaction (HER) are required due to the negative onset potential of vanillin (≈ − 0.6 V vs. RHE). As these materials are mostly toxic, such as Pb, Hg or Cd, our group investigated different non-toxic cathode materials showing that Zn cathodes can be used for hydrovanilloin production with neglectable amounts of vanillyl alcohol and high faradaic efficiencies in alkaline aqueous media [22, 23].

**Scheme 1:**
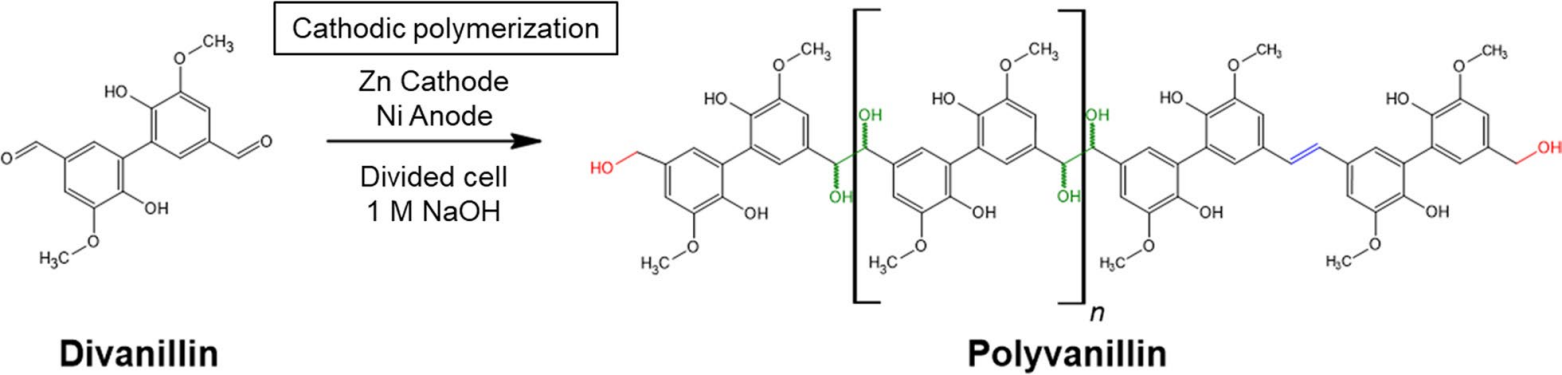
Electrochemical reduction of divanillin to polyvanillin

Divanillin (5,5-bis-vanillyl) is an easily accessible compound from vanillin bearing two remote carbonyl groups, which is generated by enzymatic aryl-aryl coupling with either horseradish peroxidase and H_2_O_2_ [24] or laccase in O_2_-saturated solution [15]. Interestingly, utilizing the same reaction type of electrochemical pinacolization a molecular weight increase from divanillin to polyvanillin is possible, which was first described by Amarasekara et al. in a feasibility study at Pb cathodes in a divided beaker cell in 2012 [25]. Recently, our group further investigated structural features of the formed polyvanillin by size exclusion chromatography (SEC) and 2D-NMR (HSQC, ^13^C/^1^H) in H-type batch cells at Zn, Pb and GC cathodes. Thereby, we showed that the molecular weight increase by electrochemical pinacolization is analogously to the vanillin reduction competing with alcohol formation, which terminates the polymer chain. Further, we found stilbene-like double bound systems in the aliphatic region of polyvanillin (Scheme 1). After complete carbonyl consumption molecular weights of M_w_ = 3200 g mol^−1^ and M_n_ = 2400 g mol^−1^ versus pullulan standard were reached for Zn cathodes showing no significant influence of current density or divanillin concentration in the H-type Batch cell [22].

Since electrosyntheses in H-type batch cells often show poor performance due to a sluggish mass transport, wide electrode distances resulting in large ohmic losses and no uniform potential distribution [26], we herein report for the first time the polyvanillin synthesis by electrochemical divanillin pinacolization in a plane parallel flow reactor in recirculation mode. The transfer onto an electrochemical flow reactor enables us to present deeper insights into the reaction mechanism by using online analytics in the electrolyte loop offering access to high time resolved data. Further, defined flow conditions and uniform current distributions in the flow reactor allows precise investigation of the impact of the current density on structural features of produced polyvanillin samples, which we analyzed by SEC and 2D-NMR (HSQC, ^13^C/^1^H). Lastly, we show an approach using dimensionless numbers for reaching reasonable high current densities of > 50 mA cm^−2^ at higher divanillin concentration and calculate the corresponding key figures of merit, such as space–time-yield *STY* and specific energy consumption *E*_s_ [27]. The resulting polymer is then thermally characterized by TGA and DSC analysis.

## Materials and methods

### Chemicals

All aqueous solutions were prepared with ultrapure water (0.055 µS cm^−1^). Sodium hydroxide (Carl Roth, p.a., ≥ 98%), 1 M hydrochloric acid (Carl Roth, standard solution/p.a.), vanillin (Sigma Aldrich, reagent plus, 99%) and pyridine-d_5_ (Sigma Aldrich, for NMR spectroscopy, min. 99.8% D) were used as received. Divanillin was synthesized as described in a previous publication [22]. Zn sheets (HMW Hauner GmbH & Co. KG, Germany, 99.99%) and Ni sheets (HMW Hauner GmbH & Co. KG, Germany, 99.99%) were used as cathode and anode material, respectively.

### Flow reactor setup and experiments

A divided plane parallel flow reactor in recirculation batch mode was used. The setup and the reactor are described in detail in a previous publication [23]. Briefly, catholyte and anolyte chamber of the reactor were separated by a Nafion N324 membrane and flat electrodes with an electrode area of 4 × 14 cm^2^ each were used. The distance between each electrode and the separator was 0.5 cm. Catholyte and anolyte were fed parallel to the electrode surface into the reactor. Inhouse 3D-printed (Photon S, Anycubic) inert plastic mesh turbulence promoters made of acrylate-based UV curing resin (Value DLP Resin, PrimaCreator) covering the full reaction channel above each electrode (4 × 14 cm^2^) were inserted in both electrolyte chambers to enhance the mass transport within the reactor and to prevent the membrane from bulging. Both turbulence promoters had a mesh width of 5 × 5 mm^2^ in diagonal orientation with respect to the flow direction. The cathodic turbulence promoter was made of 6 layers with a web thickness of 1 mm each resulting in an overall voidance of 0.7, whereas the anodic turbulence promoter was made of 4 layers with a web thickness of 1.1 mm each resulting in an overall voidance of 0.72. For further detailed investigation of the mass transport behavior obtained for the turbulence promoters see our previous publication (type D cathode side and type C anode side) [23]. Catholyte and anolyte were each circulated from a glass reservoir through the reactor by a gear pump (VGS 24 V OEM, Verder Deutschland GmbH & Co. KG). The mean linear flow velocities in the reactor were measured by flow meters (FCH-midi-PCDF, B.I.O.-Tech e.K.) positioned before the reactor entries. A reversible hydrogen reference electrode (RHE, HydroFlex^©^, Gaskatel) was connected in flow-by mode to the catholyte chamber by a 1/16′′ PTFE tube. Thereby, the PTFE tube was fixated within the turbulence promotor and the end was positioned as close as possible to the cathode to minimize the iR-drop. A minimum electrolyte flow was established and the withdrawn electrolyte was fed back catholyte reservoir. A Bio-Logic SAS SP-150 potentiostat coupled with a VMP3 10 A booster was used for performing all electrochemical measurements.

Before each measurement a Zn sheet and a Ni sheet, serving as cathode and anode respectively, were polished with SiC papers with decreasing roughness (FEPA #P180/#P500/#P1000, Struers GmbH, Germany) and rinsed with ethanol and water. The Nafion N324 membrane was soaked in 1 M NaOH for at least 24 h before the experiment to ensure its Na^+^-form. The catholyte was freshly prepared by dissolving 7.55 g (45.30 g for the high concentration experiment) of divanillin in 0.5 l of 1 M NaOH resulting in a 50 mM (300 mM) divanillin solution. The anolyte consisted of 1 l of 1 M NaOH. The volume ratio of anolyte to catholyte was 2:1 to maintain a sufficient high ionic conductivity, since Na^+^ ions migrate from the anolyte through the Nafion membrane to the catholyte in the electrolysis. Catholyte and anolyte were recirculated before starting the electrolysis at the targeted mean linear flow velocity of 20 cm s^−1^ until a steady state flow behavior set in, which was usually 5 to 10 min.

Linear sweep voltammograms (LSVs) of both the cathodic and anodic reaction were performed at a potential sweep rate of 3.33 mV s^−1^. Anode and cathode side were changed to record the anodic LSV to minimize the distance between the working and the reference electrode. The recorded potentials were corrected by the iR-drop of the electrolyte solution between the working and the reference electrode. The iR-drop was determined by galvano impedance spectroscopy (GEIS) measured at a current of -9 mA cm^−2^ and an amplitude of 0.9 mA cm^−2^ between frequencies of 100 kHz and 10 Hz.

Bulk electrolysis was performed galvanostatically at current densities of 5, 9 and 18 mA cm^−2^ for the 50 mM divanillin solution and at 54 mA cm^−2^ for the 300 mM divanillin solution until a charge of 8 F mol^−1^ passed. The experiment at 9 mA cm^−2^ was conducted three times to evaluate the overall experimental error. The reaction was monitored by an online UV–VIS setup. The resulting polymer was isolated after the electrolysis by acidifying the catholyte to pH ≈ 2 with 1 M HCl. An overview of the isolated yields is shown in the supporting information (Additional file 1: Table S1). The precipitate was filtered, thoroughly washed with water and dried in a desiccator with silica gel under vacuum overnight. The isolated polymer was then analyzed by SEC and 2D-NMR (HSQC, ^13^C/^1^H). Further, aliquots of few milliliters each were withdrawn from the catholyte throughout the electrolysis to monitor the polymerization. The sampling was divided into two experiments to minimize the amount of withdrawn catholyte and, therefore, the impact onto the reaction. The withdrawn aliquots were isolated similar to the bulk catholyte with the difference of using a 12 mL syringe with an inserted filter paper for the filtration and washing step due to its small amount.

### Investigation of Zn behavior after resting phase at open circuit potential

To access the behavior of Zn cathodes after resting at open circuit potential (OCP) accompanied investigation were conducted in an undivided beaker cell. 50 mL of 1 M NaOH were filled in a glass beaker. A 2 × 2.5 cm^2^ Zn-piece and a 1.5 cm^2^ Pt-piece were used as working and counter electrode, respectively. A reversible hydrogen reference electrode (RHE, HydroFlex^©^, Gaskatel) was used as reference electrode. The Zn electrode was allowed to rest in OCP (≈ -450 mV vs. RHE) for 10 min. Afterwards, 3 cyclic voltammogram (CV) cycles between − 0.450 V *vs.* RHE and − 1.1 V *vs.* RHE were recorded with a potential sweep rate of 20 mV s^−1^. The procedure comprised of 10 min OCP resting phase followed by 3 CV cycles was repeated 3 times. Potentials were corrected after the experiment by the iR-drop between the working and the reference electrode determined by potentio electrochemical impedance spectroscopy (PEIS). The iR-drop was 0.4 Ohm.

### Rotating disc electrode (RDE) experiments

RDE experiments were conducted to determine the diffusion coefficient of divanillin. A Pb disc (Ø_disc_ = 5 mm, Pine Research) was inserted in a PTFE tip holder (E6R1 Change Disk RRDE, Pine Research). The Pb surface was polished with a 0.05 µm diamond suspension (Buehler), rinsed with water and sonicated in ultra-pure water for at least 10 min. The RDE was then mounted in an electrode rotator (MSR Rotator, Pine Research). The electrochemical measurements were conducted in a standard three electrode setup and a BioLogic SAS SP-150 potentiostat was used. A freshly prepared 20 mM Divanillin in 1 M NaOH solution served as electrolyte, which was deoxygenated with argon gas for at least 30 min before the measurement. A platinated Pt sheet and a reversible hydrogen electrode (RHE Hydroflex^©^, Gaskatel, Germany) were used as counter and reference electrode, respectively. The potential of the RDE was held at − 0.50 V vs. RHE and then CVs between − 0.50 V vs. RHE and − 1.10 V vs. RHE were recorded at a potential sweep rate of 10 mV s^−1^ for different rotation rates (100, 400, 900, 1600, 2500 rpm). The recorded potentials were corrected by the iR-drop after the measurement, which was determined by PEIS measurement at − 0.80 V vs. RHE with an alternating potential of 10 mV and frequencies between 10 Hz and 200 kHz. The diffusion coefficient was obtained by applying the Levich-equation to the limiting current densities *j*_lim_ extracted at a potential of -0.9 V vs. RHE:

1 $${j}_{{\text{lim}}}=0.62n{\text{F}}c{D}^\frac{2}{3}{\nu }^{-\frac{1}{6}}{\omega }^\frac{1}{2}$$

where *n* is the number of transferred electrons, F is the Faraday constant (96485 As mol^−1^), *c* is the divanillin concentration, *D* is the diffusion coefficient, ν is the kinematic viscosity and ω is the angular velocity. A value of *n* = 2 was assumed, as the one electron reduction of each carbonyl group to the pinacol is expected to be the main reaction pathway in 1 M NaOH at Pb cathodes at moderate negative potentials of − 0.9 V vs. RHE [21, 22]. The experiment was conducted two times and the resulting mean value of the diffusion coefficient was calculated.

### Online UV–VIS setup

**Fig. 1:**
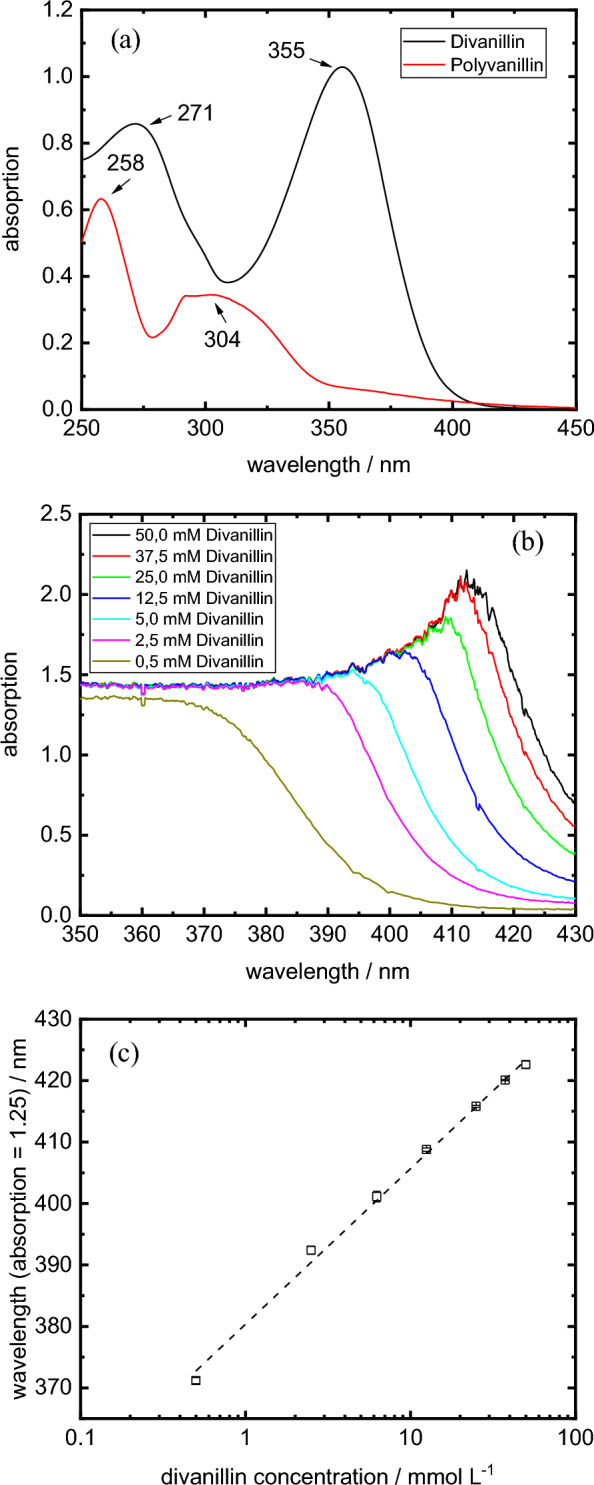
**a** UV–VIS spectrum of divanillin (7.8 µM) and polyvanillin (equivalent mass to divanillin) in 1 M NaOH recorded on a UV-1650PC Spectrometer (Shimadzu). **b** Exemplary calibration spectra of high concentrated divanillin solutions (1 M NaOH) in the online UV–VIS setup. **c** Semi-logarithmic fit of the calibration line in the online UV–VIS setup. Fit function: wavelength [nm] = 380.37 nm + 25.31 nmL/mmol * c_divanillin_ [mmol L^−1^]. R^2^ = 0.9975

The electrochemical consumption of divanillin was monitored by an online UV–VIS setup. A similar setup was used to track the vanillin electroreduction to hydrovanilloin in a previous publication in the same reactor setup [23]. The online UV–VIS setup was implemented in a bypass in the catholyte loop. It consisted of an all-quartz glass flow through cuvette (1 mm optical path length, Hellma GmbH & Co. KG), a cuvette holder (CUV-UV/VIS, Avantes), a deuterium tungsten halogen light source (DT-Mini-2-GS, Ocean Optics) and an UV–VIS spectrometer (USB 2000 + , Ocean Optics). Divanillin exhibits an absorption peak at 355 nm, which corresponds to the absorption of its carbonyl group. Upon the electroreduction of divanillin to polyvanillin the peak is vanishing (Fig. 1a). For comparison the structural similar compound vanillin exhibits an absorption peak of its carbonyl group at 348 nm, where both the one-electron reduction product hydrovanilloin and the two-electron reduction product vanillyl alcohol do not absorb [23]. As the setup measures directly the undiluted catholyte in the bypass, the spectrometer quickly reaches its detection limit. Therefore, the flank of the peak was used for evaluation. The calibration of the UV–VIS system was conducted with divanillin solutions with concentrations between 0.5 mM and 50 mM (Fig. 1b). For higher concentrations the sensitivity of the system was not sufficient enough. A linear correlation was found in the semi-logarithmic plot of the divanillin concentration versus the wavelength at an absorption of 1.25 (Fig. 1c). The calibration was carried out three times from freshly prepared solutions. In case of the 300 mM divanillin reduction experiment online UV–VIS measurement was not feasible due to the high absorption of divanillin resulting in low precision and sensitivity. Therefore, aliquots were withdrawn at distinct time intervals from the catholyte and after a 1 to 6 dilution with water the samples were offline measured at the UV–VIS setup leading to a lower amount of data points in the high concertation experiment.

### Size exclusion chromatography (SEC)

The molecular weight distributions (MWD) of the isolated polyvanillin samples were measured at a LC-system (LC 1200, Agilent Technologies) coupled with a refraction index detector using three different columns (PSS MCX 10 µm as guard column, 10 µ, 100 Å and 10 µ, 1000 Å as analytical columns) according to the literature [28]. 2–3 mg of isolated polyvanillin were dissolved in 1 mL 0.1 M NaOH solution. 20 µL of the solution were injected into the system. The mobile phase was 0.1 M NaOH. A flow rate of 1 mL min^−1^ and a temperature of 35 °C was set. Conventional pullulan standards (342 g mol^−1^ to 805,000 g mol^−1^, PSS Mainz, Germany) were used for calibration. Therefore, molecular weights are presented versus pullulan.

### 2D-NMR (HSQC, ^13^C/.^1^H)

2D-NMR (HSQC, ^13^C/^1^H) spectra were recorded on a 500 MHz Bruker AVANCE spectrometer. 50 mg of isolated and vacuum dried polyvanillin were dissolved in 600 µL Pyridin-d_5_. The samples were fully dissolved, if not stated otherwise. Chemical shifts are given in ppm downfield form TMS (δ = 0.00). The spectra were adjusted to the δ_C_/δ_H_ cross coupling signals of pyridine-d5 (δ_H_ in ppm/δ_C_ in ppm: 7.220/123.87; 7.580/135.91; 8.740/150.35). The generated 2D-NMR spectra were processed on a MestReNova Software.

### Thermogravimetric analysis (TGA)

TGA of selected samples were performed on a TG 209 F1 Iris (Nietzsch). Approximately 8 mg of the sample were heated from 25 to 950 °C in an alumina crucible with a heating rate of 10 K min^−1^ under synthetic air atmosphere (flow rate of 20 mL min^−1^).

### Differential scanning calorimetry (DSC)

DSC analyses of selected samples were performed on a DSC 1 device (Mettler Toledo). Approximately 2 mg of the sample were weighed in a high-pressure crucible with gold sealing. The sample was heated under N_2_ atmosphere with a heating rate of 10 K min^−1^ from 25 to 300 °C, cooled down to 25 °C and heated up again to 300 °C.

## Results and discussion

### Polarization curve

**Fig. 2:**
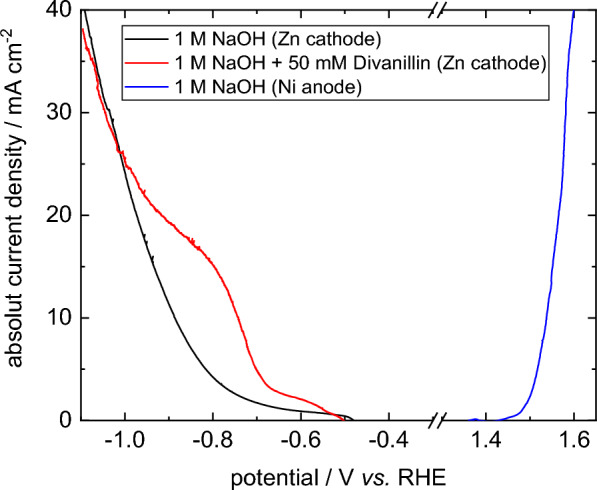
LSV of the HER at the Zn cathode in the blank catholyte (black), at the Zn cathode with addition of 50 mM divanillin to the catholyte (red) and of the OER at the Ni anode (blue). Potentials were corrected by the iR-drop. Potential sweep rate: 3.33 mV s^−1^. Mean linear flow rate: 20 cm s^−1^

First, we recorded linear sweep voltammograms (LSVs) of the blank catholyte and anolyte solutions as well as with addition of 50 mM divanillin to the catholyte to get familiar with the electrochemical system (Fig. 2). In the blank electrolytes the hydrogen evolution reaction (HER) and the oxygen evolution reaction (OER) occur at the cathode and anode side, respectively. We observed an exponential increase of the current density with increasing overpotential showing, as expected, no mass transport limitation of both reactions in the investigated current density region. When adding divanillin to the catholyte solution a broad reduction peak occurs with an onset potential of the divanillin reduction at ≈ − 675 mV vs. RHE. At potentials more negative than − 0.9 V vs. RHE the polarization curve approaches the HER at current densities of ≈ 20 mA cm^−2^. We observed parasitic currents in the potential region between − 500 mV and − 650 mV vs. RHE, which are probably due to the electroreduction of an oxide layer on the Zn electrode building up in the OCP in the start-up phase until a steady-state hydrodynamic behavior sets. The OCP of the Zn cathode in 1 M NaOH was ≈ − 450 mV vs. RHE. The value of the parasitic currents varied between ≈ 1–2 mA cm^−2^ depending on the length of the start-up phase, which was usually longer with addition of divanillin in the electrolyte due to initial foam formation until the system was free of gases. Therefore, smaller parasitic currents were observed in the LSV measurement in pure 1 M NaOH compared to the electrolyte with addition of divanillin. To support the suggestion of the formation of an oxide layer on the Zn electrodes in the OCP, accompanied CV studies of Zn electrodes in an undivided beaker cell in 1 M NaOH were performed (Additional file 1: Fig. S1). Three CV cycles between − 0.45 V vs. RHE and − 1.1 V vs. RHE after a resting phase of 10 min in OCP were recorded showing a cathodic peak at ≈ − 500 mV for the first cycle, which vanishes in the two consecutive CV cycles. As other reduction processes of the electrolyte besides the HER in pure 1 M NaOH can be excluded, this peak is attributed to the reduction of a formed oxide layer on the Zn electrode. The elimination of this peak after the first CV cycle is properly explained by the reduction of the Zn surface after cathodic polarization. The formation of this layer was confirmed by repeating this experiment three times showing a peak in the first cycle of ≈ 1–2 mA cm^−2^ at ≈ − 500 mV vanishing in the consecutive cycles.

### RDE studies and limiting current density determination

In the next step, we conducted RDE studies to determine the diffusion of coefficient of divanillin in order to calculate the limiting current densities of the divanillin reduction in the flow reactor system. Exemplary CVs of the divanillin reduction at different rotations rates and the corresponding Levich-plot in the limiting current region are shown in the supporting information (Additional file 1: Fig. S2). We observed a similar onset potential at the Pb RDE compared to the Zn cathode in the flow reactor of ≈ − 675 mV vs. RHE for the divanillin reduction. Comparatively, the cathodic onset potential of the structural similar compound vanillin at Zn and Pb were also similar [22]. No parasitic currents occurred as the OCP of Pb is slightly more positive in 1 M NaOH (≈− 200 mV), no start-up phase was needed and the potential was held at -0.5 V vs. RHE before the actual CVs. A mean value of 4.07 ± 0.09*10^–6^ cm^2^ s^−1^ for the diffusion coefficient of divanillin in 1 M NaOH is calculated from two measurements. We determined a slightly higher diffusion coefficient for vanillin in 1 M NaOH of 6.85*10^–6^ cm^2^ s^−1^, which agrees with divanillin being a larger compound than vanillin [23].

Applying the dimensionless hydrodynamic characterization of the flow reactor from our previous study [23], the limiting current density *j*_lim_ within the flow reactor system can be calculated for a given mean linear flow velocity according to the following equations:

2 $$Sh=1.83R{e}^{0.38}S{c}^{0.33}= \frac{{k}_{{\text{m}}}{d}_{{\text{e}}}}{{D}_{{\text{Divanillin}}}}$$

3 $${j}_{{\text{lim}}}=n{\text{F}}{k}_{{\text{m}}}c$$

where *Sh* is the Sherwood number, *Re* is the Reynolds number, *Sc* is the Schmidt number, *k*_m_ is the mass transport coefficient, *d*_e_ is the hydrodynamic diameter, *D*_Divanillin_ is the diffusion coefficient of divanillin and c is the bulk concentration of divanillin in the catholyte. A limiting current density of ≈18 mA cm^−2^ is calculated for a divanillin concentration of 50 mM and a mean linear flow velocity of 20 cm s^−1^ (for detailed calculation see Additional file 1). It should be mentioned that the limiting current density should decrease for oligomers building up in the electroreduction of divanillin to polyvanillin, as the diffusion coefficient decreases with increasing molecular weight [29], which is not covered in here.

### Galvanostatic electrolysis of divanillin

#### General synthesis

We performed galvanostatic electrolysis of the divanillin solution in the flow reactor. Galvanostatic electrolysis was chosen over potentiostatic electrolysis, since a future scale-up is facilitated and no potential measurement of the working electrode is necessary [26]. As drawback, the selectivity of the reaction is controlled only indirectly, as the potential of the working electrode adjusts correspondingly to the applied current and the reaction conditions. However, as introduced by others[30–32], the dimensionless current density γ, which is defined as the fraction of the applied current density *j* in relation to the limiting current density at *t* = 0:

4 $$\gamma = \frac{j}{{j}_{{\text{lim}},\mathrm{ t}=0}}$$

readily predicts the reaction outcome and can be used as a measure for the overpotential in galvanostatic electrolysis.

**Fig. 3:**
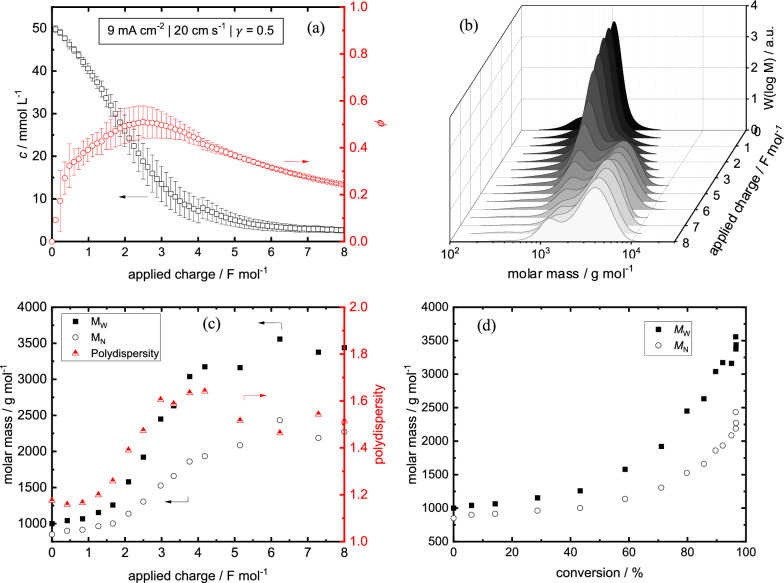
Exemplary courses of **a** substrate concentration measured by online UV–VIS and faradaic efficiency (assuming 100% pinacolization) vs. charge, **b** molecular weight distributions measured by SEC vs. charge, **c** weight averaged and number averaged molar mass and polydispersity vs. charge, **d** weight averaged and number averaged molar mass vs. conversion measured by online UV–VIS. Parameters: 50 mM initial divanillin concentration, 9 mA cm^−2^, 20 cm s^−1^ and γ = 0.5. Error bars indicate standard deviations of 3 experiments up to 4 F mol^−1^ and 4 experiments up to 8 F mol.^−1^

As first step for the galvanostatic electrolysis, we selected a moderate dimensionless current density of γ = 0.5 at a start divanillin concentration of 50 mM. The concentration course of divanillin and the faradaic efficiency course for the consumption of divanillin is shown in Fig. 3a, assuming one electron reduction of its carbonyl groups to the corresponding pinacols. It should be noted that the concentration course of divanillin is measured by the UV absorption peak of divanillin’s carbonyl groups. Consequently, carbonyl groups in terminal positions of oligomeric and polymeric reaction intermediates resulting from mono-pinacolized divanillin should be also covered by the online UV–VIS measurement. Since γ is less than 1, we expected a kinetic controlled phase at the beginning of the electrolysis, which would then transition to mass transport control with increasing consumption of divanillin’s carbonyl groups. This would be expressed by a linear decrease and an exponential decrease of the substrate concentration with time (or applied charge in galvanostatic electrolysis) for the kinetic and the mass transport phase, respectively. However, we observed a slower non-linear decrease of the substrate concentration in the kinetic controlled region, which suggests a pronounced conditioning phase of the Zn cathode. Also, this is shown in the course of the faradaic efficiency, where a steep increase of the faradaic efficiency at the start of the electrolysis is observed until a constant faradaic efficiency sets in. We strongly suspect this due to the electroreduction of the zinc oxide layer building up in the start-up phase, where the cathode remains in the OCP, as discussed above. At applied charges higher than ≈ 3 F mol^−1^ the mass transport limitation sets in resulting in an exponential decrease of the substrate concentration and the faradaic efficiency. At an applied charge of > 6 F mol^−1^ full conversion of divanillin’s carbonyl groups is achieved showing no further concentration decrease. The final concentration measured by the online UV–VIS setup is not zero, which is suspected to be from the overlapping UV–VIS absorption spectra of the produced polyvanillin and divanillin.

The molecular weight increase due to C–C coupling by pinacolization of divanillin substrate measured by SEC is shown in Fig. 3b and c. The molecular weight distribution (MWD) of the resulting polyvanillin exhibits a bimodular distribution, which agrees with obtained data from the polyvanillin synthesis in the batch cell [22]. Thereby, a small prepeak is observed at lower molecular weights of ≈1200 g mol^−1^ and a larger main peak at higher molecular weights of ≈ 4100 g mol^−1^. Final weight averaged molecular weights of M_w_ = 3588 ± 344 g mol^−1^ and number averaged molecular weights M_n_ = 2207 ± 139 g mol^−1^ are achieved after 8 F mol^−1^. The calculated polydispersity of 1.5 shows a relatively narrow MWD. No further increase of the molecular weight is observed after an applied charge of 6 F mol^−1^, which matches the concentration course of divanillin measured by online UV–VIS. Figure 3d shows the relationship of the divanillin conversion versus the molecular weight. The plot resembles an exponential increase of M_w_ and M_n_ with increasing divanillin conversion, whereby higher molecular weights are achieved only at the end of the electrolysis at high substrate conversions. This relationship suggests a step-growth polymerization producing mainly smaller molecules, such as dimers and trimers at low divanillin conversions. As the electrolysis progresses C–C coupling of molecules of different degrees of polymerization can occur leading to a rapid increase of M_w_ and M_n_. However, the polymerization finally reaches a plateau and no further increase is observed with ongoing electrolysis. To explain the latter, we recorded 2D-NMR (HSQC, ^13^C/^1^H) spectra of a partly and a full polymerized sample at an applied charge of 2 and 8 F mol^−1^, respectively (Additional file 1: Fig. S5). The assignment of the peaks to the corresponding structural features of polyvanillin is described in detail in our previous study, where a similar structure for polyvanillin was obtained in the batch cell [22]. As expected, we observed no remaining aldehyde groups after an applied charge of 8 F mol^−1^, which agrees with the UV–VIS and the SEC data. If no aldehyde groups remain after 8 F mol^−1^ and pinacolization would be the only occurring reaction, very large molecular weights should be observed. However, two electron reduction of the aldehyde group to the corresponding alcohol instead of C–C coupling by pinacolization terminates a further polymer chain growth. The alcohol production should mainly occur at the end of the electrolysis, when the carbonyl concentration is low and the radical dimerization is less likely to appear due to low local radical concentrations. Moreover, with decreasing divanillin concentration the working electrode potential is shifting toward more negative values in galvanostatic electrolysis. These more negative potentials also lead to an increase of alcohol production instead of pinacolization [21]. However, in general the significant increase of the molar mass from divanillin to polyvanillin can be seen from broadening of the peaks in the ^1^H and ^13^C spectra at an applied charge of 8 F mol^−1^ in comparison 2 F mol^−1^, e.g. see methoxy groups at δ_H_ = 3.7 ppm/δ_C_ = 55.5 ppm or the aromatic ring systems at δ_H_ ≈ 7–8 ppm/δ_C_ = 105–130 ppm. Further, we could confirm the already known stilbene-like double bound systems in the aliphatic regions of polyvanillin obtained in our batch cell experiments for the flow cell electrolysis. Their exact formation mechanism is still a subject of further studies, although similar stilbene-like structural features were achieved from acetylated pinacol groups and epoxides in the presence of Zn [14, 33, 34].

#### Impact of current density

**Fig. 4:**
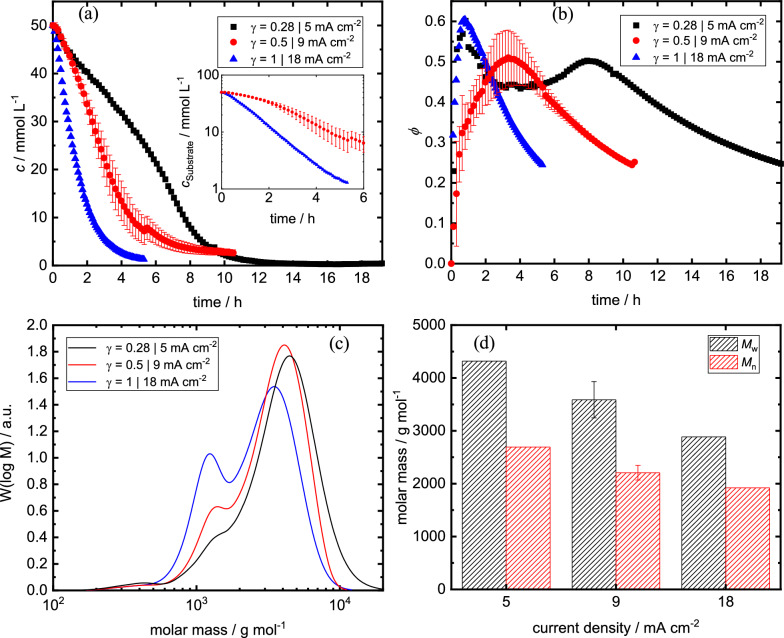
Impact of current density on **a** concentration courses, **b** faradaic efficiency (assuming 100% pinacolization) courses, **c** molecular weight distributions and **d** weight-averaged and number averaged molar masses at end of electrolysis. Inlaid plot in **a** shows concentration course of the 9 and 18 mA cm^−2^ experiments on a logarithmic y-axis. Parameters: 50 mM initial divanillin concentration, 20 cm s^−1^, 8 F mol^−1^

As a next step, we were interested in the impact of the current density on the concentration courses and on structural features of the resulting polymer polyvanillin. Figure 4a and b show the concentration profiles of divanillin and the faradaic efficiency for its conversion versus the reaction time for the three different applied dimensionless current densities γ = 0.28, 0.5 and 1 corresponding to a current density of 5, 9 and 18 mA cm^−2^, respectively. Thereby, the applied charge of 8 F mol^−1^ and the mean linear velocity of 20 cm s^−1^ were held constant. For the sake of completeness concentration profiles of divanillin and the faradaic efficiency plotted against the applied charge are shown in the supporting information (Additional file 1: Fig. S3 and Fig. S4). Surely, we observed a faster conversion with increasing current density for γ ≤ 1, as a kinetically controlled phase in the electrolysis is expected. For the highest current density of 18 mA cm^−2^ the reaction is mass transport limited almost from the beginning of the electrolysis, which agrees with the calculated limiting current density at the given reaction conditions. For example, the semi-log plot of the divanillin concentration versus time shows a linear behaviour (inlaid plot in Fig. 4a) and the faradaic efficiency decreases exponentially with time. However, the start of the electrolysis is also at 18 mA cm^−2^ overlaid by a suggested conditioning phase of the cathode, even if only little. In detail, the conditioning phase gets less pronounced with increasing current density, which would be explained by a decrease of the current ratio in the cathode conditioning in relation to the total applied current.

Figure 4c and d show the impact of the current density on the MWD and on the achieved M_w_ and M_n_ values for polyvanillin after full divanillin conversion at an applied charge of 8 F mol^−1^. We observed a significant decrease of M_w_ and M_n_ with increasing current density. This agrees with the suggestion that at higher current densities alcohol formation increases due to more negative potentials at the cathode leading to an earlier polymer chain termination. This suggestion was confirmed by 2D-NMR (HSQC, ^13^C/^1^H) analyses of the polyvanillin synthesized at 9 and 18 mA cm^−2^. A significant increase of the terminal alcohol peak was observed in the 2D-NMR spectra (Additional file 1: Fig. S6 and Table S2). Further, the amount of stilbene-like double bound groups also increases with increasing current densities suggesting that more severe reaction conditions promote also the stilbene formation. Interestingly, with decreasing current density the prepeak at low molar masses in the MWD is decreasing. This leads to the suggestion that the bimodular distribution of the molar mass would be originated from the competing alcohol formation of the carbonyl reduction. However, as the MWD of divanillin is overlapping with the prepeak, we cannot make any statement about the time of the actual prepeak formation.

When comparing these results to polyvanillin synthesized in an H-type batch cell in our previous study, slightly higher molecular weights were achieved in the flow cell. Moreover, we observed no significant impact of the current density on M_w_ and M_n_ in the batch cell, e.g. decreasing only from M_w_ = 3171 g mol^−1^ at 15 mA cm^−2^ to M_w_ = 3016 g mol^−1^ at 60 mA cm^−2^ for a Zn cathode after an applied charge of 8 F mol^−1^ and a divanillin concentration of 100 mM [22]. One possible explanation for this could be the more inhomogeneous distribution of the reactant concentration and the current density along the electrode’s surface compared to the homogenous distribution in a symmetrically-shaped plane parallel flow reactor. Therefore, batch cells often lack reproducibility, as the orientation of the working electrode is geometrically non-equivalent with regard to the counter electrode [26]. As a result, at certain sites of the electrode in the batch cell depending on their orientation more severe potentials and current densities, as expected from average distribution, can occur leading to unwanted reaction outcomes. Hereby, alcohol formation may occur on these cathode sites, e.g. at the backside of the working electrode, and lower molecular weights are achieved. Moreover, the sensitivity of impact parameter, such as the current density, become less pronounced. Higher molecular weights of polyvanillin synthesized in the flow cell and a significant impact of the current density on the MWD would agree with this suggestion.

#### Increasing the divanillin start concentration

**Fig. 5:**
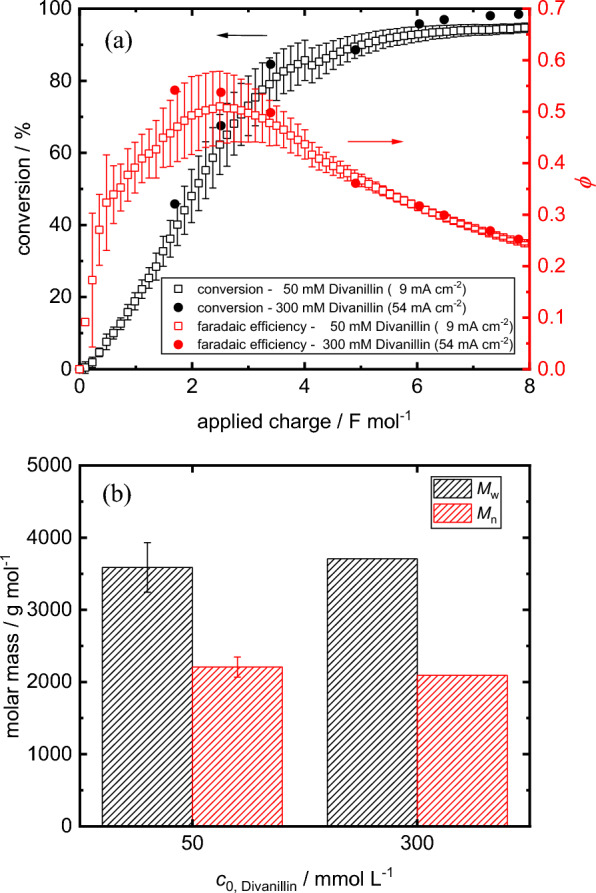
Impact of initial divanillin concentration on **a** conversion and faradaic efficiency courses and **b** weight-averaged and number averaged molar masses. Parameters: 9 mA cm^−2^ for 50 mM divanillin and 54 mA cm^−2^ for 300 mM divanillin, γ = 0.5, 20 cm s^−1^

The productivity of polyvanillin is capped by the limiting current density of the divanillin reduction. As seen from Eq. (3), the limiting current density can be increased by increasing either the mass transport coefficient or the bulk concentration of divanillin. As already high flow velocities of 20 cm s^−1^ within the reactor are present and a further increase would lead to high pressure drops over the flow reactor, we increased the start concentration of divanillin by a factor of 6 to 300 mM instead. Thereby, we held the dimensionless current density constant at a value of γ = 0.5 expecting a similar reaction outcome. Figure 5a shows the conversion of divanillin and the faradaic efficiency course for the consumption of divanillin for the experiment conducted at a high divanillin concentration of 300 mM and a current density of 54 mA cm^−2^. For comparison the reference experiment with the same dimensionless current density of γ = 0.5 at a low divanillin concentration of 50 mM and a current density of 9 mA cm^−2^ is shown. The conversion as well as the corresponding faradaic efficiency courses of the 54 mA cm^−2^ attempt lie within the error bars of the 9 mA cm^−2^ experiment and, therefore, equal conversion behaviour of divanillin is confirmed. No significant change of the viscosity of the catholyte at the end of the reaction was measured in the high concentration attempt. Further, we observed similar resulting M_w_ and M_n_ values of polyvanillin after an applied charge of 8 F mol^−1^ synthesized at 300 mM and 50 mM divanillin concentration (Fig. 5b). However, the MWDs of both samples are slightly different showing a slightly higher prepeak at ≈ 1200 g mol^−1^ and a broader main peak at the same peak maxima of 4100 g mol^−1^ (Additional file 1: Fig. S7). This difference may be explained by a higher local radical concentration in the vicinity of the cathode at higher substrate concentration and current densities favoring the dimerization step. Consequently, fractions of higher molecular weights can be expected in the MWD of polyvanillin synthesized at high concentrations. At the end of the electrolysis more severe potential for the 54 mA cm^−2^ experiment at the cathode occur, as the potential is dominated by the HER in the mass transport controlled region of the divanillin reduction. As a result, the alcohol formation leading to a more pronounced prepeak should be increased at more negative cathode potentials. However, more studies need to be conducted to confirm this suggestion. Moreover, increasing the starting concentration lead to a significant increase of the isolated yields of polyvanillin after the electrolysis from ≈ 52–57% for 50 mM to 94% for 300 mM (Additional file 1: Table S1). This increase is well explained by the remaining solubility of polyvanillin or low-molecular-weight oligomers within the product in the acidified catholyte. The high isolated yields confirm that polyvanillin is the major product in the electrolysis and potential by-products may only be produced in very small amounts. However, the remaining filtrate after the polyvanillin separation was not analyzed. Overall, we observed equal behaviour of the divanillin reduction at a constant dimensionless current density when increasing the start concentration in terms of conversion rates and faradaic efficiencies, but the reaction outcome is slightly different, which may be due to the complex behaviour of the reductive electrochemical polyvanillin formation.

We calculated key figures of merit, such as the space–time-yield *STY* and the specific energy consumption *E*_s_, for the divanillin conversion to evaluate the productivity of the polyvanillin synthesis as follows:

5 $$STY(t)=\frac{{c}_{0, Divanillin}{V}_{t}X\left(t\right){M}_{Divanillin}}{{V}_{R}t}$$

6 $${E}_{s}(t)=\frac{{\int }_{0}^{t}I{U}_{cell}\left(t\right)dt}{{c}_{0, Divanillin}{V}_{t}X\left(t\right){M}_{Divanillin}}$$

where *c*_0,Divanillin_ is the start concentration of divanillin, *V*_t_ is the volume of the catholyte in the glass reservoir, *X* is the conversion of divanillin, *M*_Divanillin_ is the molar mass of divanillin (302 g mol^−1^), *V*_R_ is the volume of the catholyte within the reactor, *t* is the reaction time, *I* is the total current and *U*_cell_ is the voltage between cathode and anode. Table 1 summarizes the *STY* and the *E*_s_ values of the low and high concentration experiment. The reaction courses of the *STY* and *E*_s_ for both experiments at low and high concentration for γ = 0.5 are shown in the supporting information (Additional file 1: Fig. S8). As expected, a six-fold increase of the *STY* from 0.072 kg l^−1^ h^−1^ to 0.471 kg l^−1^ h^−1^ in the kinetically controlled region was achieved when increasing the divanillin concentration from 50 to 300 mM, whereby *E*_s_ only increased by 27% from 0.938 kWh kg^−1^ to 1.191 kWh kg^−1^. Thereby, the moderate increase of *E*_s_ results from an increase of the mean cell voltage from 2.58 V to 3.63 V due to higher overpotentials for the HER and the OER at higher current densities. The overpotential for the actual divanillin reduction should remain equal at a constant dimensionless current density and, therefore, would not contribute to the increase of the cell voltage. At high conversions of e.g. 90% the *STY* decreases to 0.314 kg l^−1^ h^−1^ and the *E*_s_ values increases to 1.787 kWh kg^−1^ for the 300 mM experiment, as more and more charge is consumed by the competing HER as soon as the divanillin reduction becomes mass transport limited. For comparison, maximum *STY* of 1.13 kg l^−1^ h^−1^ for the vanillin reduction to the hydrodimer hydrovanilloin and 1.18 kg l^−1^ h^−1^ for L-cysteine hydrochloride synthesis were published for similar flow reactor systems in recirculation mode at low substrate conversions at the start of the electrolysis [23, 35]. *E*_s_ values under the same reaction conditions for the hydrovanilloin and L-cysteine hydrochloride synthesis were 0.46 and 1.6 kWh kg^−1^, respectively. Moreover, Table 2 compares the performance of this study with the previous studies investigating polyvanillin synthesis in divided batch cells. As expected, a significant improvement of the space–time-yield and the specific energy consumption was obtained in the flow cell compared to previous batch cell approaches. The improvements can be attributed to higher electrode area to volume ratios, better mass transport and more narrow distances between the electrodes resulting in faster conversion and lower cell voltages impacting these key figures of merit. Lastly, it should be noted that fivefold higher molecular weights were obtained by Amarasekara et al. [25] compared to our studies in batch [22] and flow. However, no calibration standards were stated and the SEC analysis was performed in DMF, which makes the results hardly comparable.

**Table 1:** Impact of high concentration and high current density attempt on the molar mass of synthesized polyvanillin and on key figures of merit for performance evaluation

Current density (mA cm^−2^)	Divanillin concentration (mM)	γ	M_w_ (g mol^−1^)	M_n_ (g mol^−1^)	Mean cell voltage (V)	*STY* in kinetic zone^a^ and (at X = 90%) (kg l^−1^ h^−1^)	*E*_s_ in kinetic zone^a^ and (at X = 90%) (kWh kg^−1^)
9	50	0.5	3588 ± 344	2207 ± 139	2.58 ± 0.02	0.072 ± 0.010 (0.052 ± 0.002)	0.938 ± 0.137 (1.277 ± 0.042)
54	300	0.5	3709	2094	3.63	0.471 (0.314)	1.191 (1.787)

Parameters: 20 cm s^−1^, 8 F mol^−1^

Error bars indicate standard deviations of at least 3 experiments

^a^After conditioning phase of the electrode

**Table 2:** Comparison of the performance of divanillin reduction of this study with the literature

Cathode	Catholyte	Electrochemical parameters	Cell configuration	Applied charge/ F mol^−1^	Molecular weight/ g mol^−1^	*STY*^d^/ kg l^−1^ h^−1^	*E*_s_^d^/ kWh kg^−1^	Refs.
Zn	0.3 M divanillin + 1 M NaOH	Galvanostatic 54 mA cm^−2^	Divided flow reactor in recirculation mode	8	M_w_ = 3709^b^ M_n_ = 2094^b^	0.206	2.72	This study
Zn	0.1 M divanillin + 1 M NaOH	Galvanostatic 60 mA cm^−2^	H-type batch cell (50 mL catholyte)	8	M_w_ = 3016^b^ M_n_ = 2201^b^	0.006	5.70^e^	[22]
Zn	0.3 M divanillin + 1 M NaOH	Galvanostatic 60 mA cm^−2^	H-type batch cell (50 mL catholyte)	8	M_w_ = 3208^b^ M_n_ = 2344^b^	0.007	6.62^e^	[22]
Pb	0.175 M divanillin + 1 M NaOH	Galvanostatic using 12 V supply (1.1 A for 3 h)^a^	Divided beaker cell (20 mL catholyte)	17.6	M_w_ = 16066^c^ M_n_ = 10729^c^	0.016	40.91^f^	[25]

^a^Current density cannot be stated, as immersed area of electrodes (2.5*9 cm^2^) in the electrolyte was not given

^b^Versus pullulan standard

^c^Avarage from 6 polymerization trials. No calibration standard stated (SEC performed with DMF as solvent)

^d^Calculated using isolated yield of polyvanillin after electrolysis. ^e^Avarage cell voltage of 6.5 V

^f^Assuming a cell voltage of 12 V (power supply)

### TGA and DSC analysis of polyvanillin

**Fig. 6:**
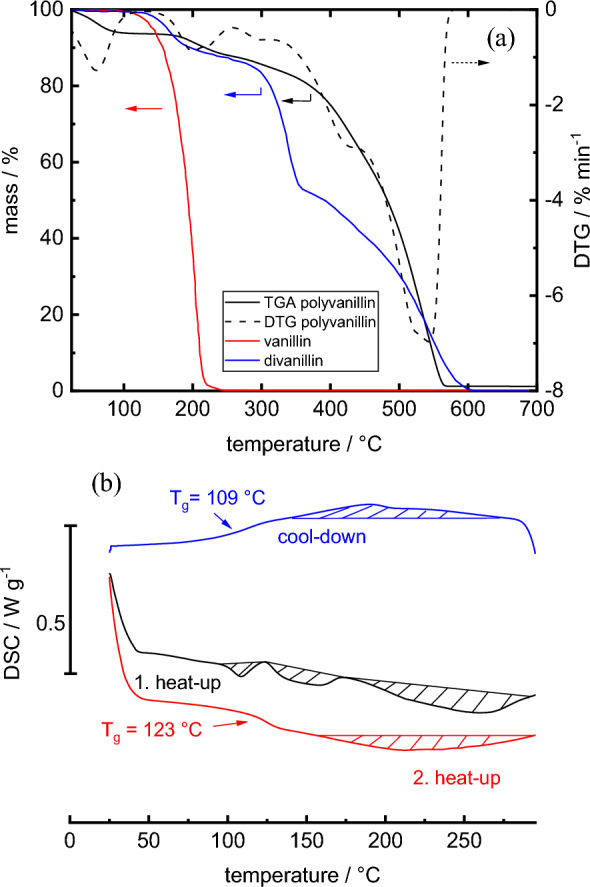
TGA (**a**) and DSC (**b**) analysis of polyvanillin. For comparison TGAs of vanillin and divanillin are also shown. Reaction conditions polyvanillin: 54 mA cm^−2^, 8 F mol^−1^, 300 mM divanillin, γ = 0.5, 20 cm s^−1^

Finally, TGA and DSC analysis were conducted of polyvanillin produced at 54 mA cm^−2^ (Fig. 6a and b). The TGA shows that the polymer exhibits good thermal stability with a mass loss of 50% at 484 °C. However, beside the actual decomposition of the polymer at an onset temperature of 322 °C and a decomposition temperature of 541 °C (referring to the minimum peak temperature of the DTG curve), we observed two more mass losses below 100 °C and at ≈ 200 °C. We suggest that the first and second mass losses are due to residual moisture or crystal water and lower molecular compounds within the polymer or from their cleavage from the polymer chain, respectively. The second mass loss could also be from H_2_O cleavage of the pinacol groups of polyvanillin, however, vanillin and divanillin exhibiting thermal degradation at ≈200 °C indicate loss of vanillin derivatives. Neglecting the first two mass losses and setting the onset decomposition temperature of the polymer at 322 °C as reference point, a temperature of 5% decomposition of 371 °C is determined.

The DSC analysis shows no glass transition temperature in the first heat-up phase. However, we observed complex behavior with three no sharp thermal transitions at peak temperatures of 109 °C, 146 °C and 253 °C, respectively. Cooling down the melt led to reorganization of molecules in a broad zone with a peak temperature of 190 °C and a glass transition temperature of *T*_g_ = 109 °C was observable. In the second heat-up phase a glass transition temperature of *T*_g_ = 123 °C followed by a broad melting phase at a peak temperature of 212 °C was measured. These results indicate thermal behavior of a thermoplastic material, with properties like glass transition as well as melting and crystallization.

No glass transition temperature and a slightly lower 50% weight loss in the TGA of 440 °C was found for polyvanillin by Amarasekara et al. in their batch cell feasibility study [25]. However, the ^1^H-NMR analysis exhibited sharp peaks and presence of still unreacted terminal aldehyde groups was observed indicating no full polymerization. The transfer onto a flow reactor with improved mass transport in the present study compared to the batch cell led to full polymerization of divanillin, which could explain the difference in the thermal characterization of polyvanillin.

Comparatively, Llevot et al. found for a totally divanillin-based polyester (corresponding to polymer P7 in the publication) a glass transition temperature of *T*_g_ = 102 °C with a 50% weight loss at *T* ≈ 475 °C [15]. The phenolic groups of divanillin were methylated before the polymerization process. No melting transition was found between − 70 and 200 °C. The absence of crystallinity in the divanillin-based polyester in contrast to polyvanillin could be due to the absence of phenolic groups resulting in amorphous polymers. When comparing with commercially available polymers exhibiting one glass transition and one melting temperature, polyvanillin shows most similar thermal behavior to polyphenylene sulfide (PPS). PPS exhibits a glass transition temperature of *T*_g_ = 90 °C, a melting temperature of ≈ 285 °C and a decomposition temperature of ≈ 530 °C [36].

## Conclusion

We systematically studied the synthesis of polyvanillin by electrochemical pinacolization of divanillin at a Zn cathode in a divided plane parallel flow reactor in recirculation mode. Prevenient calculation of the limiting current density for the divanillin reduction using the hydrodynamic characterization of the flow reactor and the diffusion coefficient of divanillin enabled us to select suitable galvanostatic reaction parameters. We used the dimensionless current density γ as figure of merit for the reaction outcome and to estimate kinetic and mass transport limitations. We showed a charge resolved molecular weight increase from divanillin to polyvanillin, whereby full divanillin conversion was reached after an applied charge of ≈ 6 F mol^−1^. Despite the negative onset potential of divanillin of − 650 mV vs. RHE, faradaic efficiencies of 50–60% were reached in the kinetically controlled region due to the high HER overpotential at Zn cathodes. A plot of divanillin conversion against the molecular weight of the product revealed a step-growth polymerization for the polyvanillin synthesis, whereby the competing 2e^−^ reduction to the corresponding alcohol terminates the chain growth and caps the maximum final molecular weight of polyvanillin. This was confirmed by 2D-NMR (HSQC, ^13^C/^1^H) showing besides expected pinacol and terminal alcohol groups also stilbene-like double bounds in the aliphatic region in the resulting polymer confirming the structure of polyvanillin synthesized in previous batch studies [22]. The molecular weight of polyvanillin decreased with increasing current densities after complete consumption of carbonyl groups in divanillin due to an increasing formation of terminal alcohol groups. Lastly, we were able to increase the productivity of polyvanillin synthesis by a sixfold increase of the divanillin start concentration. While maintaining a constant dimensionless current density, the electrolysis was conducted at current densities of 54 mA cm^−2^ for a divanillin start concentration of 300 mM divanillin showing similar conversion behavior compared to the 50 mM experiment at 9 mA cm^−2^. Thereby, promising space–time-yields up to 0.47 kg l^−1^ h^−1^ at specific energy consumptions of 1.19 kWh kg^−1^ could be reached for the polyvanillin production, in which averaged molecular weights of M_w_ = 3700 g mol^−1^ and M_n_ = 2100 g mol^−1^ were achieved. The characterization of polyvanillin by TGA and DSC analysis revealed good thermal stability and thermal behavior of a thermoplastic material with *T*_g_ = 109–123 °C, *T*_melting_ = 190–212 °C and *T*_decom._ = 541 °C.

### Supplementary Information

**Additional file 1. **Isolated yields of polyvanillin, additional CVs of Zn cathode after resting phase in OCP, CVs of RDE experiments, detailed calculation of the limiting current density, conversion and Faradaic efficiency plots versus charge of the impact of the current density, 2D-NMR spectra of the polyvanillin samples and results of selected peak integrations as well as additional data of the high divanillin concentration attempt. **Table S1.** Isolated yield of polyvanillin after acidification of the catholyte to pH = 2 electrolyte. **Table S2.** Methoxy group normalized ^1^H-peak areas of the terminal alcohol and stilbene groups of the 2D-NMR spectra. Parameters: 50 mM initial divanillin concentration, 8 F mol^−1^, 20 cm s^−1^. **Figure S1.** CVs of Zn-cathode in 1 M NaOH after a resting phase of 10 min each in OCP. Four consecutive rounds were conducted, which are indicated by the round number displayed in the corresponding graph. Parameters: Undivided beaker cell (50 mL of electrolyte – 1 M NaOH), RT, WE: 5 cm^2^ Zn-piece, CE: 1.5 cm^2^ Pt-piece, REF: RHE (Fa. Gaskatel), CVs are corrected by iR-drop (0.4 Ohm determined via PEIS), 3 cycles -0.45 V vs. RHE to -1.1 V vs. RHE with 20 mV s^−1^. **Figure S2.** **a** Exemplary CVs of divanillin in 1 M NaOH measured at the RDE on a Pb disc with a potential sweep rate of 10 mV s^−1^. **b** Levich-plot for determination of the diffusion coefficient of divanillin in 1 M NaOH. Limiting current densities were extracted at − 0.9 V vs. RHE. **Figure S3.** Impact of current density on concentration courses. Inlaid plot in shows concentration course of the 9 and 18 mA cm^−2^ experiments on a logarithmic y-axis. Parameters: 50 mM initial divanillin concentration, 20 cm s^−1^, 8 F mol^−1^. **Figure S4.** Impact of current density on Faradaic efficiency courses (assuming 100% pinacolization). Parameters: 50 mM initial divanillin concentration, 20 cm s^−1^, 8 F mol^−1^. **Figure S5.** 2D-NMR (HSQC, ^13^C/^1^H) spectra of isolated polyvanillin after an applied charge of **a** 2 F mol^−1^, which partly precipitated, and **b** 8 F mol^−1^. Parameters: 50 mM initial divanillin concentration, 9 mA cm^−2^, 20 cm s^−1^ and γ = 0.5. Solvent was pyridin-d_5_. **Figure S6.** 2D-NMR (HSQC, ^13^C/^1^H) spectra of isolated polyvanillin after an applied charge of 8 F mol^−1^ synthesized at a current density of **a** 9 mA cm^−2^ and **b** 18 mA cm^−2^. Parameters: 50 mM initial divanillin concentration, 20 cm s^−1^. Solvent was pyridin-d_5_. **Figure S7.** Molecular weight distributions of the low and high concentration experiment. Parameters: 20 cm s^−1^. **Figure S8.** Space–time-yields *STY* and specific energy consumption *E*_S_ courses for the high concentration (300 mM) and high current density (54 mA cm^−2^) experiment. For comparison the low concentration (50 mM) and low current density (9 mA cm^−2^) experiment of the same dimensionless current density of γ = 0.5 is shown. Parameters: 20 cm s^−1^.

## Data Availability

All data generated or analyzed during this study are included in this published article (and its additional information files).

## References

[1] Schobert H (2013). Chemistry of fossil fuels and biofuels. Cambridge series in chemical engineering.

[2] Sudesh K, Iwata T (2008). Sustainability of biobased and biodegradable plastics. Clean.

[3] Fache M, Auvergne R, Boutevin B (2015). New vanillin-derived diepoxy monomers for the synthesis of biobased thermosets. Eur Polym J.

[4] Miller J (2021) Lignin 2021: A pivotal year. Biofuels Digest

[5] Zirbes M, Waldvogel SR (2018). Electro-conversion as sustainable method for the fine chemical production from the biopolymer lignin. Curr Opin Green Sustain Chem.

[6] Zirbes M, Quadri LL, Breiner M (2020). High-temperature electrolysis of kraft lignin for selective vanillin formation. ACS Sustain Chem Eng.

[7] Bawareth B, Di Marino D, Nijhuis TA (2018). Unravelling electrochemical lignin depolymerization. ACS Sustain Chem Eng.

[8] Zirbes M, Graßl T, Neuber R (2023). Peroxodicarbonate as a green oxidizer for the selective degradation of kraft lignin into vanillin. Angew Chem Int Ed.

[9] Klein J, Kupec R, Stöckl M (2023). Degradation of lignosulfonate to vanillic acid using ferrate. Adv Sustain Syst.

[10] Klein J, Alt K, Waldvogel SR (2022). Selective degradation of lignosulfonate and lignin with periodate to 5-iodovanillin. Adv Sustain Syst.

[11] Fache M, Boutevin B, Caillol S (2015). Vanillin, a key-intermediate of biobased polymers. Eur Polym J.

[12] Chauhan NPS (2014). Preparation and characterization of bio-based terpolymer derived from vanillin oxime, formaldehyde, and p-hydroxyacetophenone. Des Monomers Polym.

[13] Koike T (2012). Progress in development of epoxy resin systems based on wood biomass in Japan. Polym Eng Sci.

[14] Harvey BG, Guenthner AJ, Meylemans HA (2015). Renewable thermosetting resins and thermoplastics from vanillin. Green Chem.

[15] Llevot A, Grau E, Carlotti S (2015). Renewable (semi)aromatic polyesters from symmetrical vanillin-based dimers. Polym Chem.

[16] Frontana-Uribe BA, Little RD, Ibanez JG (2010). Organic electrosynthesis: a promising green methodology in organic chemistry. Green Chem.

[17] Pearl IA (1952). Reactions of vanillin and its derived compounds. XVI the synthesis of vanillil. J Am Chem Soc.

[18] Amarasekara AS, Hasan MA (2016). Vanillin based polymers: III. Electrochemical dimerization of vanillin revisited and synthesis of hydrovanilloin–formaldehyde polymer. Polym Sci Ser B.

[19] Amarasekara AS, Garcia-Obergon R, Thompson AK (2019). Vanillin-based polymers: IV. Hydrovanilloin epoxy resins. J Appl Polym Sci..

[20] Amarasekara AS, Garcia-Obregon R (2021). Vanillin based polymers: V. Poly (hydrovanilloin–urethane). Polym from Renew Resour.

[21] Jow J-J, Choi TC (1987). Product distributions and kinetics of cathodic reduction of vanillin in aqueous solution. Electrochim Acta.

[22] Kunkel R, Schmidt VM, Cremers C (2021). Electrochemical synthesis of biobased polymers and polymer building blocks from vanillin. RSC Adv.

[23] Kunkel R, Kovács MM, Müller D (2021). Electrochemical vanillin reduction in a plane parallel flow reactor: characterization, modeling and process improvement. Electrochim Acta.

[24] Nishimura RT, Giammanco CH, Vosburg DA (2010). Green, enzymatic syntheses of divanillin and diapocynin for the organic, biochemistry, or advanced general chemistry laboratory. J Chem Educ.

[25] Amarasekara AS, Wiredu B, Razzaq A (2012). Vanillin based polymers: I. An electrochemical route to polyvanillin. Green Chem.

[26] Pletcher D, Green RA, Brown RCD (2018). Flow electrolysis cells for the synthetic organic chemistry laboratory. Chem Rev.

[27] Kunkel R (2022) PhD Thesis. Wissenschaftliche Schriftenreihe des Fraunhofer ICT. Karlsruhe Institute of Technology

[28] Baumberger S, Abaecherli A, Fasching M (2007). Molar mass determination of lignins by size-exclusion chromatography: towards standardisation of the method. Holzforschung.

[29] Moacanin J, Felicetta VF, Haller W (1955). Lignin. VI. molecular weights of lignin sulfonates by light scattering 1. J Am Chem Soc.

[30] Guena T, Pletcher D (1998). Electrosyntheses from aromatic aldehydes in a flow cell. Part I. The reduction of benzaldehyde. Acta Chem Scand.

[31] Galia A (2007). Electrochemical Synthesis of d, l -homocysteine thiolactone hydrochloride in a batch continuous recirculation reactor equipped with carbon felt cathodes: a study for the optimization of the process. Ind Eng Chem Res.

[32] Robinson D (1990). Electrosynthesis—from laboratory to pilot to production.

[33] He B, Tian H, Geng Y (2008). Facile synthesis of 9,10-diarylphenanthrenes and poly(9,10-diarylphenanthrene)s. Org Lett.

[34] Huang J-M, Lin Z-Q, Chen D-S (2012). Electrochemically supported deoxygenation of epoxides into alkenes in aqueous solution. Org Lett.

[35] Ralph TR, Hitchman ML, Millington PJ (2005). The importance of batch electrolysis conditions during the reduction of L-cystine hydrochloride. J Electrochem Soc.

[36] Schindler A, Doedt M, Gezgin Ş (2017). Identification of polymers by means of DSC, TG, STA and computer-assisted database search. J Therm Anal Calorim.

