# Supplementary material for: Electroreduction of divanillin to polyvanillin in an electrochemical flow reactor
Source: BMC Chem. 2024 Feb 8;18(1):28. doi: 10.1186/s13065-024-01133-2 (PMC10854100; doi:10.1186/s13065-024-01133-2)
Supplement: Supplementary file 1 — Additional file 1. Isolated yields of polyvanillin, additional CVs of Zn cathode after resting phase in OCP, CVs of RDE experiments, detailed calculation of the limiting current density, conversion and Faradaic efficiency plots versus charge of the impact of the current density, 2D-NMR spectra of the polyvanillin samples and results of selected peak integrations as well as additional data of the high divanillin concentration attempt. Table S1. Isolated yield of polyvanillin after acidification of the catholyte to pH = 2 electrolyte. Table S2. Methoxy group normalized 1H-peak areas of the terminal alcohol and stilbene groups of the 2D-NMR spectra. Parameters: 50 mM initial divanillin concentration, 8 F mol−1, 20 cm s−1. Figure S1. CVs of Zn-cathode in 1 M NaOH after a resting phase of 10 min each in OCP. Four consecutive rounds were conducted, which are indicated by the round number displayed in the corresponding graph. Parameters: Undivided beaker cell (50 mL of electrolyte – 1 M NaOH), RT, WE: 5 cm2 Zn-piece, CE: 1.5 cm2 Pt-piece, REF: RHE (Fa. Gaskatel), CVs are corrected by iR-drop (0.4 Ohm determined via PEIS), 3 cycles -0.45 V vs. RHE to -1.1 V vs. RHE with 20 mV s−1. Figure S2. a Exemplary CVs of divanillin in 1 M NaOH measured at the RDE on a Pb disc with a potential sweep rate of 10 mV s−1. b Levich-plot for determination of the diffusion coefficient of divanillin in 1 M NaOH. Limiting current densities were extracted at − 0.9 V vs. RHE. Figure S3. Impact of current density on concentration courses. Inlaid plot in shows concentration course of the 9 and 18 mA cm−2 experiments on a logarithmic y-axis. Parameters: 50 mM initial divanillin concentration, 20 cm s−1, 8 F mol−1. Figure S4. Impact of current density on Faradaic efficiency courses (assuming 100% pinacolization). Parameters: 50 mM initial divanillin concentration, 20 cm s−1, 8 F mol−1. Figure S5. 2D-NMR (HSQC, 13C/1H) spectra of isolated polyvanillin after an applied charge of a 2 F mol−1, which part [file 13065_2024_1133_MOESM1_ESM.docx]

**Electroreduction of Divanillin to Polyvanillin in an Electrochemical Flow Reactor – Supporting Information**

Robin Kunkel*^,1^, Maximilian Fath^1,2^, Detlef Schmiedl^3^, Volkmar M. Schmidt^2^, Jens Tübke^1^

^1^Fraunhofer Institute for Chemical Technology ICT, Department of Applied Electrochemistry, Joseph-von-Fraunhofer-Str. 7, D-76327 Pfinztal, Germany.

^2^Mannheim University of Applied Sciences, Institute of Chemical Process Engineering, Paul-Wittsack-Str. 10, D-68163 Mannheim, Germany

^3^Fraunhofer Institute for Chemical Technology ICT, Department of Environmental Engineering, Joseph-von-Fraunhofer-Str. 7, D-76327 Pfinztal, Germany

*Corresponding author: robin.kunkel@ict.fraunhofer.de

ORCID:

Robin Kunkel - 0000-0001-5285-2369

Detlef Schmiedl – 0000-0001-7335-6145

Jens Tübke – 0000-0003-3317-2637

**Isolated yields of polyvanillin**

**Table S1** Isolated yield of polyvanillin after acidification of the catholyte to pH = 2 electrolyte.

| Current density (mA cm^-2^) | c_divanillin_ (mM) | Applied charge (F mol^-1^) | Isolated yield (%)^a^ |
| --- | --- | --- | --- |
| 5 | 50 | 8 | 52 |
| 9 | 50 | 8 | 55 |
| 18 | 50 | 8 | 57 |
| 54 | 300 | 8 | 93 |

^a^Calculation based on weight.

**Additional CVs of Zn cathode after resting phase in OCP**

**Fig. S1** CVs of Zn-cathode in 1 M NaOH after a resting phase of 10 minutes each in OCP. Four consecutive rounds were conducted, which are indicated by the round number displayed in the corresponding graph. Parameters: Undivided beaker cell (50 mL of electrolyte – 1 M NaOH), RT, WE: 5 cm^2^ Zn-piece, CE: 1.5 cm^2^ Pt-piece, REF: RHE (Fa. Gaskatel), CVs are corrected by iR-drop (0.4 Ohm determined *via* PEIS), 3 cycles -0.45 V vs. RHE to -1.1 V vs. RHE with 20 mV s^-1^.

**CVs of RDE experiments**

**Fig. S2** (a) Exemplary CVs of divanillin in 1 M NaOH measured at the RDE on a Pb disc with a potential sweep rate of 10 mV s^-1^. (b) Levich-plot for determination of the diffusion coefficient of divanillin in 1 M NaOH. Limiting current densities were extracted at -0.9 V vs. RHE

**Calculation of the limiting current density**

The limiting current density for the divanillin reduction was calculated from the dimensionless characterization of the mass transport of our previous study.^[1]^ Therefore, the Reynolds number *Re* and Schmidt number *Sc* were first calculated as follows:

$$Re=\frac{vd_{e}}{\nu}=\frac{0.2 m s^{-1}*0.0089 m}{1.38*{10}^{-6} m^{2} s^{-1}}=1290$$

$$Sc= \frac{\nu}{D_{Divanillin}}=\frac{1.38*{10}^{-6} m^{2}s^{-1}}{4.07*{10}^{-10} m^{2}s^{-1}}=3391$$

where *v* is the mean linear velocity in the catholyte chamber, *d*_e_ is the hydrodynamic diameter (for calculation see previous study^[1]^), ν is the kinematic viscosity of the catholyte (value corresponds to the supporting electrolyte 1 M NaOH and impact on ν of divanillin was neglected) and *D*_Divanillin_ is the diffusion coefficient of divanillin determined at the RDE.

The Sherwood number Sh is then calculated as follows

$$Sh=1.83Re^{0.38}Sc^{0.33}=407.$$

Lastly, the mass transport coefficient for the divanillin reduction *k*_m_ and the limiting current density for the start concentration 50 mM divanillin is derived:

$$k_{m}=\frac{{ShD}_{Divanillin}}{d_{e}}=\frac{407*4.07*{10}^{-10} m^{2}s^{-1}}{0.0089 m}=1.86*{10}^{-5} m s^{-1}$$

$$j_{\lim}=nFk_{m}c=2*96485 As mol^{-1}*1.86*{10}^{-5} m s^{-1}*0.05*{10}^{3} mol m^{-3}=17.95 mA cm^{-2}$$

where *n* is the number of transferred electrons, F is the Faraday constant and *c* is the divanillin concentration. A value of *n* = 2 was assumed, as the one electron reduction of each carbonyl group to the pinacol is expected to be the main reaction pathway in 1 M.^[2]^

**Impact of current density – conversion and Faradaic efficiency plots vs. charge**

**Fig. S3** Impact of current density on concentration courses. Inlaid plot in shows concentration course of the 9 and 18 mA cm^-2^ experiments on a logarithmic y-axis. Parameters: 50 mM initial divanillin concentration, 20 cm s^-1^, 8 F mol^-1^

**Fig. S4** Impact of current density on Faradaic efficiency courses (assuming 100% pinacolization). Parameters: 50 mM initial divanillin concentration, 20 cm s^-1^, 8 F mol^-1^

**2D-NMR spectra of isolated polyvanillin samples**

**Fig. S5** 2D-NMR (HSQC, ^13^C/^1^H) spectra of isolated polyvanillin after an applied charge of (a) 2 F mol^-1^, which partly precipitated, and (b) 8 F mol^-1^. Parameters: 50 mM initial divanillin concentration, 9 mA cm^-2^, 20 cm s^-1^ and 𝛾 = 0.5. Solvent was pyridin-d_5_

**Fig. S6** 2D-NMR (HSQC, ^13^C/^1^H) spectra of isolated polyvanillin after an applied charge of 8 F mol^-1^ synthesized at a current density of (a) 9 mA cm^-2^ and (b) 18 mA cm^-2^. Parameters: 50 mM initial divanillin concentration, 20 cm s^-1^. Solvent was pyridin-d_5_

**Table S2** Methoxy group normalized ^1^H-peak areas of the terminal alcohol and stilbene groups of the 2D-NMR spectra. Parameters: 50 mM initial divanillin concentration, 8 F mol^-1^, 20 cm s^-1^

| **Current density  [mA cm^-2^]** | **Normalized alcohol peak of the ^1^H-spectra  (δ_H_ = 5.0 ppm)**  $\frac{\boldsymbol{A}_{\boldsymbol{terminal OH groups}}}{\boldsymbol{A}_{\boldsymbol{Methoxy groups}}}$ | **Normalized stilbene peak of the ^1^H-spectra  (δ_H_ = 6.7 ppm)**  $\frac{\boldsymbol{A}_{\boldsymbol{Stilbengroups}}}{\boldsymbol{A}_{\boldsymbol{Methoxy groups}}}$ |
| --- | --- | --- |
| 9 | 0.017 | 0.011 |
| 18 | 0.030 | 0.025 |

**Additional data of the high concentration attempt**

**Fig. S7** Molecular weight distributions of the low and high concentration experiment. Parameters: 20 cm s^-1^

**Fig. S**8 Space-time-yields STY and specific energy consumption E_S_ courses for the high concentration (300 mM) and high current density (54 mA cm^-2^) experiment. For comparison the low concentration (50 mM) and low current density (9 mA cm^-2^) experiment of the same dimensionless current density of 𝛾 = 0.5 is shown. Parameters: 20 cm s^-1^

**References**

[1] R. Kunkel, M. M. Kovács, D. Müller, V. M. Schmidt, F. Simmat, J. Tübke, *Electrochim. Acta* **2021**, *391*, 138923.

[2] a) J.-J. Jow, T. C. Choi, *Electrochim. Acta* **1987**, *32*, 311; b) R. Kunkel, V. M. Schmidt, C. Cremers, D. Müller, D. Schmiedl, J. Tübke, *RSC Adv.* **2021**, *11*, 8970.
